# “You can’t have a PrEP program without a PrEP Coordinator”: Implementation of a PrEP panel management intervention

**DOI:** 10.1371/journal.pone.0240745

**Published:** 2020-10-16

**Authors:** Parya Saberi, Kristin Ming, Hyman Scott, Albert Liu, Wayne Steward

**Affiliations:** 1 Department of Medicine, University of California, San Francisco, San Francisco, CA, United States of America; 2 Bridge HIV, San Francisco Department of Public Health, San Francisco, CA, United States of America; Washington University in Saint Louis, UNITED STATES

## Abstract

Lack of healthcare provider knowledge, capacity, and willingness to prescribe PrEP are barriers to PrEP delivery in clinical settings. We implemented the PrEP Optimization Intervention (PrEP-OI) combining a PrEP Coordinator with an online panel management tool to assist providers with PrEP uptake, persistence, and management in 12 San Francisco Department of Public Health Primary Care Clinics. Medical directors (N = 10) identified important factors to consider prior to implementation, including shortage of clinical space for coordinators, medical mistrust, language barriers, and limited lab hours, along with the need for education of providers and staff and patient outreach. Among 110 providers who completed a baseline survey, the majority had reservations in asking about sexual practices and having conversations about PrEP. Providers reported PrEP-OI increased their efficiency and capacity to manage PrEP patients, and served as a gateway to additional services. These results highlight the promise of a provider-based intervention to improve the PrEP continuum and maximize the impact of PrEP.

## Introduction

HIV pre-exposure prophylaxis (PrEP) resulted in significant reduction in HIV acquisition in numerous studies [1–3]. However, despite data from the Centers for Disease Control and Prevention (CDC) estimating that nearly 1.2 million individuals had a PrEP indication, its coverage was as low as 18.1% in 2018 [4]. One major barrier to PrEP implementation is the lack of healthcare provider knowledge and willingness to prescribe it [5–8]. A report on the early experiences with PrEP uptake and delivery in San Francisco identified the need for increased PrEP knowledge among healthcare providers and the need for expanded PrEP access by developing interventions to facilitate PrEP delivery in clinical settings as priority steps to maximize PrEP’s public health impact [9].

Panel management has been defined as an approach to population-based care that proactively focuses on the health of patients assigned to a clinic. These strategies typically entail identifying a care gap, training staff to serve as panel managers, developing a registry and a health maintenance template, and adopting clinical practice guidelines to close care gaps. Panel management strategies using a patient coordinator have been used in numerous chronic conditions, including cancer, diabetes, cardiovascular disease, dementia, and HIV [10]. Task sharing associated with panel management has been shown to enhance the efficiency, consistency, and quality of care, along with improving health outcomes [11–13]. In prior studies, panel management strategies were associated with patients being referred to a PrEP provider and receiving a PrEP prescription [14]; earlier PrEP initiation [15]; and assisting with provision of patient education, adherence counseling, and resolution of insurance and pharmacy barriers [16].

We implemented a PrEP intervention consisting of a PrEP Coordinator and a web-based panel management tool called PrEP-Rx to assist healthcare providers from the San Francisco Department of Public Health (SFDPH) Primary Care Clinics in PrEP uptake, persistence, and management. In this paper, we report the results of formative discussions with medical directors, quantitative surveys with providers, and one-on-one qualitative interviews with providers from these study clinics on their experiences with the PrEP intervention. The purpose of these data were to examine facilitators and barriers to the initial implementation and sustainability of our PrEP panel management strategy.

## Materials and methods

### Study overview

The PrEP Optimization Intervention (PrEP-OI) study included PrEP coordination services provided by four PrEP Coordinators (PCs) plus a web-based panel management tool to augment and organize the PrEP coordination services. The PCs were designated non-clinical staff who coordinated the interaction between the patients and the healthcare teams and augmented the provider’s role to more effectively conduct PrEP panel management activities. The responsibilities of the PC were to examine electronic health records or patient registries to identify those who had tested positive for sexually transmitted infections (STIs) or receive referrals for PrEP initiation from the healthcare team; schedule appointments with patients to evaluate risk of HIV acquisition; assess the need for post-exposure prophylaxis (PEP); assist with PEP to PrEP transitions; educate on HIV risk reduction with PrEP use and other risk reduction methods; conduct baseline and quarterly lab tests (as allowed through SFDPH standard operating procedures) and any follow-ups visits (in person, by telephone, or via text messaging) in the place of or along with the provider visit; educate patients on STI self-swabbing; assess medical insurance coverage for PrEP and complete forms for prior authorization and/or patient assistance programs; counsel patients on PrEP initiation and persistence; provide PrEP adherence counseling; send PrEP prescription to provider for signature; communicate with the provider regarding the patient’s questions, side effects, and progress; and educate the patient on new PrEP medications or dosing strategies. As such, upon hire, the PC received training on HIV treatment and prevention, financial benefits navigation, PEP and PrEP initiation and follow-up counseling, required lab tests and frequency of testing, methods for evaluating medication adherence, risk reduction counseling, new PrEP medication options and dosing strategies, using electronic health records (EHR) and the web-based PrEP panel management tool, and providing unbiased patient care.

To create an efficient workflow for the PC, we created a web-based tool called PrEP-Rx [17] which had three main features: **1-** a comprehensive self-administered HIV risk assessment using an integrated survey tool; **2-** automated reminders to PCs for patient lab monitoring and follow-up visits for adherence, side effect assessment, and risk reduction counseling; and **3-** a PrEP timeline for each patient to allow PCs and providers to see a patient’s PrEP use history and upcoming visits in one snapshot. The risk assessment was created by the study team using the CDC risk index [18], information from Smith et al [19], and input from behavioral research experts at the University of California, San Francisco (UCSF) Center for AIDS Prevention Studies. PrEP-Rx also provided a list of questions for PCs to ascertain at PrEP initiation and follow-ups (e.g., assessing for acute HIV symptoms, screening for STIs, evaluating medication adherence, and reviewing need for refills), and the ability to export these answers into a medical chart note in the patient’s EHR. PrEP-Rx was created using a HIPAA-compliant Salesforce platform and refined using an iterative agile methodology [20].

PrEP-OI was initiated in 12 SFDPH primary care clinics (three clinics under one administration with overlapping healthcare providers and drop-in services for adolescents and young adults were grouped together for a total of 10 clinical sites). Clinics were randomized to start the intervention on a monthly basis starting November 2018 using a Stepped-wedge design, with all clinics randomized by September 2019, and were later continued on the intervention for a year follow-up phase (to be completed in September 2020). Details of the PrEP-OI study protocol have been published [21]. The UCSF Institutional Review Board (IRB) approved this study.

### Organizational assessments with medical directors

Before the initiation of the study, we met with each of the 10 clinical site’s medical directors in person to conduct a brief organizational assessment to inform how our program could best fit within existing workflows and to identify any unique aspects of the clinic that the PrEP-OI study should consider. We also asked about the clinic’s weekly schedule, lab hours, staffing, capacity for STI testing, and educational needs. This organizational assessment was guided by a set of specific questions, which were developed by the PrEP-OI investigative team and PCs. These prompts were used to ensure that we covered consistent content across clinics. Subsequently, content from the field notes were tabulated to facilitate comparison across clinics. In particular, we looked for areas of convergence, highlighting implementation considerations that were likely to affect the intervention across sites. In addition, we identified areas of divergence, which helped to inform facets of project operating protocols at each location. As the divergences were clinic-specific, we focused on areas of convergence for this manuscript, identifying those aspects of the findings that influenced the operations of the PrEP-OI across sites. Findings were grouped and summarized.

### Quantitative survey

Prior to initiation of PrEP-OI at each clinic, we asked 135 healthcare providers (defined as any PrEP prescribing provider such as physicians, nurse practitioners, pharmacists, etc.) to complete a brief online quantitative survey using Qualtrics (S1 File). Surveys were emailed to each clinic’s providers two months prior to onboarding the clinic.

The survey inquired about provider demographics (gender, race/ethnicity, primary profession, years providing direct patient care), number of patients on panel, number of patients receiving HIV treatment, number of patients on PrEP, team members who provide PrEP services in their practice (i.e., those who provide sexual risk reduction counseling, PrEP adherence counseling, and lab tests and monitoring), and frequency of offering risk reduction measures in the past year (e.g., frequency of asking about sexual partner(s), sex practices, condom use, etc.). We describe the sample of healthcare providers who participated in the quantitative survey using measures of central tendency (mean, standard deviation, etc.)

### Healthcare provider interviews

From November 2019 to February 2020, we conducted individual semi-structured qualitative interviews (S2 File) with healthcare providers from the 10 clinical sites. Participants were recruited purposively [22] as we sought to ensure variability in the clinic(s) where individuals worked, their professional training (e.g., physician, nurse practitioner), role within clinic (e.g., any management duties in addition to serving as a provider), and engagement with the PCs. To facilitate this objective, we first reached out to PCs to inquire about providers to interview based on their level of engagement with PrEP services. We then augmented these numbers by reaching out to all providers at the clinics to ensure all sites were represented. To facilitate participant comfort and honesty, scheduling of interviews with the providers, interviews, and analyses were conducted exclusively by study investigators and staff not engaged in delivery of PrEP coordination services. Interviews took place on a HIPAA-compliant videoconferencing platform to allow for flexibility in scheduling and recording of the interviews. Interviews lasted 40–60 minutes and verbal informed consent was received as approved by the UCSF IRB.

Using an interview guide, interview domains included **1-** provider’s experience with PrEP initiation and/or monitoring prior to PrEP-OI implementation; **2-** provider’s experience with PrEP initiation and/or monitoring during PrEP-OI implementation; **3-** PrEP-OI’s potential impact on the provider’s capacity, capability, or opportunity to prescribe PrEP; **4-** PrEP-OI’s role in changing other practices in the clinic; **5-** requested modifications of the PrEP-OI services to improve use; **6-** strategies to increase the provider’s PrEP offering and prescribing practices; and **7-** best ways to support providers when new PrEP formulations become available.

Qualitative interviews were audio-recorded, transcribed verbatim, and field notes were created from each interview. We followed the procedures of Framework Analysis [23], a type of thematic analysis for qualitative content. Two investigators coding and organizing the research data into a framework matrix, which was used to identify overarching themes and findings. The investigators discussed emerging patterns and themes, synthesized results based on participant responses, and selected exemplary quotes to further elucidate important discussion points.

## Results

### Organizational assessments with medical directors

We conducted a total of 10 assessments with medical directors from the 10 clinical sites. Across sites, informants highlighted a shortage of clinical space for the PC, medical mistrust, and language barriers as the top barriers to providing PrEP coordination services in their clinics. The need for education of the providers and staff, patient outreach, and coordination of PrEP services with lab hours were also noted as important challenges. Ideas for outreach to patients included contacting patients who had tested positive for an STI in the past using a clinic-approved script, asking nursing staff to refer patients being treated for STIs, and notifying nearby methadone clinics about the availability of PrEP services.

### Qualtrics survey

**Table 1** summarizes the demographics of the 110 (81.5%) healthcare providers from all 10 clinical sites who participated in the quantitative surveys. These individuals had a mean age of 41.8 years, were mainly female (74.5%), White (45.5%), and physicians (71.8%). Approximately 77.3% of the participants had ever prescribed PrEP and had a median of one patient on PrEP (**Table 2**). Participants reported to always or often offer HIV testing to patients who engaged in high-risk behaviors (76.4%), ask about sexual partners (71.8%), and ask about condom use (66.4%). However, among the 110 respondents, 50% always or often asked patients about their sex practices, 27.3% always or often asked about sexual partners’ HIV status and 21.8% always or often initiated a conversation about PrEP. Participants noted that the physicians and nurses were primarily responsible for providing sexual risk reduction counseling, PrEP adherence counseling, and PrEP lab testing and monitoring in their practice.

**Table 1:** Summary of provider characteristics from San Francisco Department of Public Health Primary Care Clinics who received the PrEP-OI intervention.

		N = 110
Age, mean years (SD)		41.8 (10)
Gender, N (%)		
	Female	82 (74.5)
	Male	25 (22.7)
	Non-binary	3 (2.7)
Race, N (%)		
	Asian	38 (34.5)
	African American	7 (6.4)
	Multiracial/Multicultural	10.9 (10.9)
	White	45.5 (45.5)
	Other	3 (2.7)
Latino/Hispanic Ethnicity, N (%)		8 (7.3)
Primary Profession/Role, N (%)		
	Physician	79 (71.8)
	Nurse Practitioner	21 (19.1)
	Pharmacist	6 (5.5)
	Physician Assistant	2 (1.8)
	Other	2 (1.8)
Primary Specialty, N (%)		
	Family Medicine	88 (80.0)
	Internal Medicine	10 (9.1)
	Other	10 (10.9)
Years providing direct patient care, Mean years (SD)		11.4 (8.6)
Patients currently in panel per provider, Median number (IQR)		200.0 (122.5–537.5)
Patients receiving HIV treatment per provider, Median number (IQR)		2.5 (1.0–10.0)

IQR: interquartile range; PrEP: pre-exposure prophylaxis; SD: standard deviation

**Table 2:** Frequency of offering PrEP or support for providing PrEP services prior to PrEP-OI implementation.

		N = 110
Ever prescribed PrEP, N (%)		85 (77.3)
Ever refer a patient for PrEP services, N (%)		55 (50.0)
Patients receiving PrEP on panel, Median (IQR)		1.0 (0.0–3.5)
Willing to prescribe PrEP for adolescents (13–17 years), N (%)		68 (61.8)
In the past year, N (%) of providers who “always” or “often”…		
	Offered HIV testing to patients who engage in high-risk behaviors	84 (76.4)
	Asked about sexual partner(s)	79 (71.8)
	Asked about condom use	73 (66.4)
	Offered HIV testing to patients who do not engage in high-risk behaviors	71 (64.6)
	Asked about sex practices	55 (50.0)
	Asked about sexual partners’ HIV status	30 (27.3)
	Initiated a conversation about PrEP	24 (21.8)
Who currently provides sexual risk reduction counseling in practice, N (%)*		
	Provider	84 (76.4)
	Nurse	38 (34.5)
	Counselor	8 (7.3)
	Social worker	7 (6.4)
	Off-site clinician	6 (5.5)
	No one	6 (5.5)
	No response	17 (15.5)
Who currently provides PrEP adherence counseling in practice, N (%)*		
	Provider	73 (66.4)
	Nurse	20 (18.2)
	Counselor	4 (3.6)
	Social worker	1 (0.9)
	Off-site clinician	3 (2.7)
	No one	19 (17.3)
	No response	17 (15.5)
Who currently provides PrEP lab testing and monitoring in practice, N (%)*		
	Provider	84 (76.4)
	Nurse	31 (28.2)
	Counselor	1 (0.9)
	Social worker	0 (0.0)
	Off-site clinician	2 (1.8)
	No one	7 (6.4)
	No response	17 (15.5)

IQR: interquartile range; PrEP: pre-exposure prophylaxis; SD: standard deviation

* Participants could select multiple options; therefore, percentages adds up to greater than 100%

### Healthcare provider interviews

From 11/20/19–2/7/20, we interviewed a select group of 24 providers from across the 8 clinical sites who had a mean age = 40.4 years (standard deviation [SD] = 9.9), 73.7% were female, 68.4% were White, and 68.4% were physicians. Interviewed providers noted that they had a median of four patients on PrEP (interquartile range [IQR] = 2–14). Responses to questions were summarized as such:

***1-*** *Provider’s experience with PrEP initiation and/or monitoring before the launch of PrEP-OI*: Participants noted barriers when initiating PrEP and following up with patients on PrEP prior to the start of PrEP-OI. At PrEP initiation, lack of time (due to having patients with complex comorbidities during short appointments), lack of personal PrEP knowledge (resulting in discomfort with discussing self-swabbing and sex), and pharmacy and insurance issues (e.g., prior authorizations and patient assistance programs) were noted as the main barriers (**Table 3**).

*I think clinicians may have a natural tendency to maybe avoid addressing things that they don't feel that they can really adequately address and spend time focusing on other things where we will be able to get the return on investment or bang for their buck*.

**Table 3:** Experiences with PrEP initiation and/or monitoring before and after launch of PrEP-OI.

Theme	Sub-theme	Details
**Experiences with PrEP initiation &/or monitoring** **before** **launch of PrEP-OI**	Initiation	Lack of time
		Lack of knowledge & comfort
		Lack of insurance & pharmacy issues
	Follow-up	Lack of time
		Reactive follow-up
		No clinical support to provide PrEP
		Lack of method to contact patients
**Experiences with PrEP initiation &/or monitoring** **after** **launch of PrEP-OI**	Awareness	PC is a conduit & opens lines of communication when patient is ready to start PrEP
		PC has more time to talk to patient about PrEP & provides more attention
		PC can educate patients & update providers & staff
	Screening	PC can provide risk/benefit analysis
		Patients more likely to disclose sexual practices to PC
		PC can review new risk factors
	Linkage	PC is support & resource for providers, so that they can focus on the medical issues
		PC facilitates conversations between provider & experts
		PC connects patients to provider & allows for continuity of PrEP care
		PC supports providers who are unfamiliar by sharing responsibility of patient care
		PC reduces provider worry because they know that PC is tracking patients
		PC brings importance of PrEP to forefront
	Initiation	PC helps with prior authorizations, insurance, & pharmacy issues
		Faster PrEP starts
		PC identifies & outreaches to PrEP candidates so there are more PrEP starts
		PC provides better panel management to identify those fallen out of care
		Providers do not need to refer patients to other clinics anymore
	Adherence & Retention	PC ensures med-taking & better retention in care
		PC provides quarterly management by ordering labs, helping with STI screen, refilling PrEP, sending reminders
		PC tracks & reaches out to patients due to organized & proactive follow-up, which allows for safety net to patients to prevent falling out of care
		PC outreaches via texting which increases access to patients especially those who are hard to reach

PC: PrEP Coordinator; PrEP-OI: Pre-exposure Prophylaxis Optimization Intervention; STI: sexually transmitted infection

At follow-up, time for refilling PrEP or ordering labs, referring patients to other locations due to not having clinical support to provide PrEP, and lack of method to contact patients unresponsive to telephone calls were the main barriers. Providers also talked about having a “reactive” follow-up strategy, meaning that they relied on the patient to request a refill or contact the provider before remembering to order labs, check in about adherence and side effects, or send refills. As a result, patients who would not request refills or contact the provider would be lost to follow-up.

*It feels a little irresponsible when we were providing PrEP prescriptions without that level of service and management*. *So this feels like night and day in terms of somebody monitoring follow up and reaching out to patients because if we don't*, *90% of our patients are not going to come back in after the 90 days if somebody doesn't reach out to them*.

***2-*** *Provider’s experience with PrEP initiation and/or monitoring after the launch of PrEP-OI*: In contrast, after the start of PrEP-OI, participants noted improvements across the entire PrEP continuum from PrEP awareness to adherence and retention in care (**Table 3**). Providers described the PC as a conduit between the provider and the patient with more time to provide PrEP education, screening, and counseling. The issue of task sharing was discussed by numerous providers in that, by having the PC provide specialized PrEP services, the provider could focus on the medically important issues.

*It took so many things off of my plate*. *So*, *I'm there to be able to do the medical assessment*. *She's able to do the things that don't have to be done by a physician*. *After visit work that needs to be done in order to ensure that patients are able to get the medication and take it appropriately and follow up*, *none of that has to be done by a physician*.

The assistance of the PC was associated with reduced provider anxiety and stress.

*It’s nice that [PC] has the time to sit down with them and use a phone interpreter*. *She’s not limited to the 15 minute in and out primary care provider visit*, *where we’re trying to address multiple things at once*. *That’s why it’s been so critical to have her on-site and have the texting capability*, *which we as primary care providers don’t really offer*. *I personally just feel better about it*. *Like I don’t worry that the patient’s falling through the cracks or if I prescribe this refill*. *Just knowing that [PC] is tracking them and following up with them*, *I just feel more at ease*.

Providers also noted the more efficient identification of PrEP candidates, increased ease of PrEP prescribing due to resolution of insurance and pharmacy issues, and faster PrEP starts (from 1–2 weeks to 1–2 days).

*It’s just simply much easier and much faster because the majority of my patients have [health access program] and I couldn’t simply write a prescription and send them to [pharmacy]*. *It could’ve been 1–2 weeks and now it’s pretty much immediate or next day or same day*. *There’s a huge difference in having him around*.

Providers reported that this increased efficiency achieved through the panel management strategy resulted in fewer patients falling out of care and not needing to be referred to another clinic to receive PrEP. Finally, providers noted an increase in PrEP adherence and retention due to frequent contact with the PC, receiving timely refills, and completing quarterly lab tests. This was referred to as a “proactive” follow-up (in contrast to the previously described “reactive” follow-up), which further prevented patients from falling out of care.

*Sometimes I would give people 90 days with one refill and say when you’re due for your refill*, *you need to come in*, *but I didn’t really have a good system*. *You know*, *we’re primary care so I don’t really have the bandwidth to monitor that*, *so having someone who is paying attention to that is really helpful*. *[PC] is proactive about getting them back in*. *My practice by necessity was a little bit reactive*. *So when I got the refill request*, *it would be like oh crap*, *ok it’s been 3 months*, *so now I need to call them or get somebody to call them to get them in*.

Additionally, the use of mobile telephones by the PC to send text messages to the patient further enhanced their capacity for patient access.

*It also just keeps this line of communication open*, *where the PrEP coordinator’s reaching out and maybe they [patients] don't respond for a while*. *And then seemingly out of the blue*, *they'll text back*: *‘oh*, *I really actually do want to restart PrEP or I think I actually need PEP*. *Can I come in today*?*’ So it keeps a communication channel open for folks to access when they're ready or it feels right for them*.

***3-*** *PrEP-OI’s potential impact on the provider’s capacity*, *capability*, *or opportunity to prescribe PrEP*: Providers noted that the shifts from before to after PrEP-OI had resulted in their increased capacity, capability, and opportunity to offer PrEP and manage patients on PrEP. This was accomplished mainly by decreasing barriers to PrEP initiation (faster resolution of insurance and pharmacy issues, providing patient education and building trust, developing care plans, removing the need for quarterly appointments with the provider, or patient referral to another clinic). Due to the perceived increase in efficiency, providers were more likely to want to offer PrEP.

*I think for some of our staff without a PrEP Coordinator*, *they felt like well*, *I can't really talk about PrEP or advocate for it that much because there's no one to hold it and they don't want to hold it*. *Some people*, *without a PrEP Coordinator*, *feel like ‘I don't want to open that box and then have to deal with all of these other responsibilities*.*’ So folks maybe felt a little less willing to really go there without the added support*.

Finally, similar to theme 2 (i.e., Provider’s experience with PrEP initiation and/or monitoring after the launch of PrEP-OI), providers often noted their general peace of mind and reduced anxiety due to their trust in the PC’s abilities for organized and thorough panel management.

***4-*** *PrEP-OI’s potential role in changing other practices in the clinic*: Providers noted several indirect impacts of PrEP-OI. This included a general increase in PrEP awareness because the PCs served as a constant visual reminder to keep PrEP at the forefront of the clinician’s and staff’s minds. PCs increased the provider’s comfort level with discussions about PrEP, sex practices, and STI self-swabbing.

*I would definitely say that the issue is more in the forefront of things that I think about when addressing health maintenance issues with our patients*. *I think that is true about the presence of a PrEP coordinator*, *because it breaks down so many barriers to getting this to happen*. *I don't know whether it's a psychological thing that I feel now it's easier to do and so I'm more likely to bring it up with patients*.

Additionally, the increased contact with patients in general was noted to be a gateway to the provision of other primary care services beyond PrEP, such as vaccinations and other healthcare maintenance.

*Biggest thing is getting people back in*. *Because then PrEP is the gateway for primary care and once we get somebody in*, *we can also be like*, *oh these are the other screenings that are recommended that we can do today since we’re already drawing your blood*, *so we'll do ton of vaccination*.

There was noticeably a more thorough sexual health history intake, patient and provider education on STI self-swabbing, and increased STI testing. Some providers noted that through PrEP-OI they had been educated about conducting appropriate testing for chlamydia and gonorrhea, from only urine testing to three-site screening (urine, pharyngeal, and rectal).

*I think because of [PC] and that [clinician’s] presentation*, *we probably before only tested for urine gonorrhea/chlamydia*, *but then after the presentations*: *‘you should probably be testing separately rectally and orally or pharyngeally*, *and this is how you teach patients to do it*.*’ Because number one*, *being told is one thing*, *but then having to teach patients how to do it and having that additional training and support and answering questions and making sure patients are comfortable doing that is a whole other hurdle*. *So having [PC] there has definitely helped with that*. *Because before her we probably only did urine*, *maybe vaginal*, *doubt that we did much rectal or oral/pharyngeal*, *because we had no idea how to do it*.

***5-*** *Requested modifications of the PrEP-OI services to improve use*: When asked about ways to improve the current PrEP-OI services, providers had few suggestions which mainly included strategies to increase the work hours and scope of work of the PC and increasing education for providers, clinic staff, and patients (**Table 4**). One suggestion involved the expansion of the responsibilities of the PC to include care coordination for those living with HIV.

**Table 4:** Improving PrEP-OI services and PrEP prescribing in general.

Theme	Sub-theme	Details
**How to improve the PrEP-OI services**	Increase PC capacity	Increase PC’s time at clinic to enhance coverage
		Allow for PC to be present at specialty clinics (e.g., urgent care, women’s clinic, etc.)
		Involve PC in coordinating care to patients living with HIV
	Education	Provide more staff & provider trainings
		Send PrEP email updates to providers
		Provide flyers & handouts, in different languages, to patients with education on cost & positive messaging
**How to increase PrEP prescribing**	Increasing referrals	Providers to standardize workflow to offer PrEP when informing patients of positive STI test & during STI treatment
		Provider & PC to review STI registry
		Optimizing EHR use by creating PrEP dot-phrases* for sexual health risk screening & safer sex counseling, preventative care screening & healthcare maintenance, STI treatment, & after-visit summary to patient
	Education & training	For providers on sexual health counseling with active listening & without judgment
		For providers to become aware of implicit biases & how to offer PrEP to everyone
		For providers & clinic staff on providing youth-friendly health education (e.g., picking up PrEP from pharmacy, refilling PrEP, & contacting PC or providers in case of problems & side effects)
	Normalize PrEP & decrease PrEP stigma	Providers to universally offer PrEP using a single question**
		No need for “PrEP clinic” or PrEP specialists as PrEP should be part of primary care
		Present on health equity & social justice to highlight who is getting access to PrEP
		Provider & patient education on PrEP not being just for MSM & TGWM but can be for anyone
	PrEP “captain”***	Attend provider meetings to answer question, updates on PrEP, discuss cases, etc.

EHR: electronic health record; MSM: men who have sex with men; PC: PrEP Coordinator; PrEP-OI: Pre-exposure Prophylaxis Optimization Intervention; STI: sexually transmitted infection; TGWM: transgender women who have sex with men

* Shortcuts to insert a predefined phrase that can be quickly summoned when typing an EHR note

** An example of a single question to universally offer PrEP: “Are you interested in hearing more about a pill that can prevent HIV?”

*** Another provider with more PrEP experience to educate other providers and staff and provide clinical consultations

***6-*** *Strategies to increase the provider’s PrEP offering and prescribing practices*: When asked about how to increase PrEP prescribing in general, providers noted several options such as ways of increasing referrals via offering PrEP to all patients who test positive for STIs and reviewing STI registries to identify patients who have previously tested positive for STIs (**Table 4**). Another option included the optimization of EHR use by creating dot-phrases (shortcuts to insert a predefined phrase that can be quickly summoned when typing an EHR note) as a reminder to the provider and staff to assess patient’s PrEP interest. Providers also discussed the need for further training on active listening without judgement, recognizing and reducing implicit biases, universally offering PrEP to all patients, and developing youth-friendly health education.

*Many of us have internal blind spots or biases*. *The epidemiology is primarily MSM [men who have sex with men]*, *and that doesn't mean we don't pay attention to the other folks that may benefit from PrEP*, *but it's hard to sometimes break out of those kinds of silos of thinking*. *I think trying to standardize workflows*, *like anytime somebody has chlamydia gonorrhea*, *when they come back for treatment*, *that also should be an automatic trigger for also talking about PrEP*. *It just should be routine*.

Another recurring theme included the need to normalize PrEP to decrease stigma associated with PrEP use by offering PrEP to all patients (not just cis-gender MSM and transgender women who have sex with men) using a single question (i.e., “are you interested in hearing more about a pill that can prevent HIV?”), offering PrEP as part of primary care instead of a specialty “PrEP clinic” with “PrEP specialists,” and further examining the clinic’s gender and racial/ethnic health disparities. Finally, providers discussed the need for a PrEP “captain” (i.e., another provider with more PrEP experience) to help other providers with PrEP education and clinical consultations.

*It seems like we’re disconnected in terms of our knowledge about how to go about making PrEP and PEP more universal and nothing special*, *like just a part of something we do*. *I think that’s a stigma*. *The assumptions are made*, *number one*, *you’re too old to be having sex*, *number two*, *you’re not high risk*. *To normalize it*, *it should be like what we did with HIV*, *just universal testing*, *it became part of your physical*. *We make assumptions about people and what their sexual behaviors are and I think we’re missing a big opportunity*.

***7-*** *Best ways to support providers when new PrEP formulations become available*: Finally, participants were asked about how they could best be supported to prescribe new PrEP formulations (e.g., injections, implants, etc.) upon availability. Many noted that they would like to receive algorithms and guidelines similar to those available for oral contraceptives or other chronic conditions (such as diabetes or hypertension). This algorithm would identify a basic PrEP regimen and circumstances for using alternative regimens along with advantages and disadvantages of each option. Providers requested that these guidelines be available through their EHRs, especially when requesting an electronic consultation (i.e., e-consult). In addition to training and educational opportunities for providers, other suggestions included training the PCs to support providers in this decision-making process and making the PC positions permanent.

*[PC] definitely has made life easier for the providers*, *and we very much appreciate having him around*. *He can establish a different kind of relationship with the client than we have*, *so I think it just helps primarily with adherence*, *and adherence is the most important thing as far as I’m concerned*. *I think it’s really important that that be a permanent position*. *Now it would be hard to imagine our clinic without him*. *Every clinic should have a PrEP coordinator*.

## Discussions

In this paper, we report facilitators and barriers to implementation of a PrEP panel management strategy for increasing PrEP use in a large safety-net health system. Shortage of clinical space, medical mistrust, language barriers, education of providers and staff, patient outreach, limited lab hours, limited provider time, and provider comfort with PrEP were noted to be important factors to consider prior to implementation. However, after implementation, need for increased PC’s work hours and scope of work; continuing education for providers, staff, and patients; and expansion of the responsibilities of the PC to taking a status neutral approach to HIV treatment and prevention were noted as ongoing issues to address. The factors reported pre- and post-implementation suggest that many of the gaps in PrEP implementation and potential benefits of the PrEP-OI intervention were not initially clear to providers. Providers reported that the PrEP-OI intervention addressed many of the (often unrecognized) barriers to PrEP implementation in a public health primary care setting. Similar discrepancies in PrEP implementation challenges have been reported in other health jurisdictions [24].

The lack of centralized care through a panel management strategy prior to PrEP-OI may have been responsible for the low number of patients on PrEP on the providers’ panels. A panel management strategy would help to address a number of identified barriers to PrEP initiation. Additionally, from our quantitative surveys, it is clear that providers have reservations about asking about patients’ sex practices, sexual partners’ HIV status, and initiation of conversations around PrEP.

In comparing PrEP management before and after the implementation of PrEP-OI, it was clear that the intervention had impacted the entire PrEP continuum from uptake to persistence and increased provider perceptions of efficiency, capacity, and capability to prescribe and manage PrEP. In addition to PrEP-specific benefits, PrEP-OI also provided an opportunity to engage patients in other primary care services (e.g., vaccinations and laboratory monitoring for hepatitis C), increase provider comfort with more thorough sexual health history taking, and STI testing. Therefore, it may be possible that PrEP-OI can augment responses to other epidemics, such as syphilis and hepatitis C.

In aligning with requests from providers, we are examining ways to optimize the use of EHR to increase PrEP referrals and education. This includes developing dot-phrases (shortcuts to insert pre-defined data or text into an EHR note) that providers can incorporate into their existing dot-phrases (e.g., for general healthcare maintenance, sexual health risk screening, or STI treatment and follow-up) or create new ones regarding PrEP offering using a single question (i.e., “are you interested in hearing more about a pill that can prevent HIV?”). If the patient notes their interest in hearing more, the provider and staff can notify the patient that the clinic’s PC will follow-up with them and route the patient’s chart to the PC. These dot-phrases serve as additional reminders to ask all patients receiving a general check-up and/or treatment for an STI, and can minimize the time allocation for PrEP discussions during a brief medical appointment. These EHR optimization strategies can also normalize PrEP and decrease PrEP stigma by universally asking all patients about their desire to reduce HIV risk. We are incorporating graphics on PrEP basics and various PrEP dosing strategies in different languages in the EHR for providers to use in after-visit summaries given to the patient. Our PCs are examining optimal ways of reviewing STI registries to capture those who have recently tested positive for an STI, and developing youth-friendly counseling points including how to pick up PrEP from a pharmacy, refill PrEP, and contact PC and clinic for any challenges.

Finally, with the availability of new PrEP formulations, providers requested algorithms similar to oral contraceptives, whereby a basic PrEP regimen would be identified and all options would be listed with pros and cons. We continue to provide education and updates to the PC, providers, and clinic staff, and develop education material for patients in different languages.

Historically, prior panel management strategies have used the services of a PrEP Navigator [14, 15], as opposed to a PrEP Coordinator. Although these terms have not yet been fully defined, a PrEP Navigator’s role is more in line with the navigation of health insurance benefits, risk reduction counseling, referral and linkage to a PrEP provider, or referral to other services (e.g., housing or mental health programs). In PrEP-OI [21], PCs provide the spectrum of PrEP services except signing PrEP prescriptions and consulting with patients in case of side effects or other clinical questions (i.e., activities requiring medical expertise). Therefore, in line with principles of task sharing, the vast majority of the activities associated with PrEP initiation and continuation can be achieved with the assistance of a PC. This allows for providers to be involved when medically necessary and to focus on other comorbidities and/or medical issues. We believe that the coordination of a patient’s PrEP care would be more efficient, organized, and reduce cost when involving a panel management strategy such as PrEP-OI versus solely reliant on healthcare providers. Although we did not have a cost-effectiveness component for this study, studies of patient navigation-type services for other conditions have found them to be cost-effective [25].

Implementation of the PrEP-OI intervention will likely require that a jurisdiction identify sustainable sources of support. This includes funding to pay for the coordinators, as well as personnel support to supervise the program. Additionally, technology costs are needed to maintain and update the online panel management tool. The coordinator positions are a viable potential strategy for cities and states looking to meet the “prevent” pillar of the US national Ending the HIV Epidemic Initiative [26], for which substantive federal funding is being allocated. States and cities may also be able to incorporate the positions into local revenue streams. At the level of the individual clinic, capitated funding models, for which a clinic receives a set payment per patient assigned, may provide greater flexibility and create greater efficiencies than other insurance models because they incentivize delivery of services that prevent diseases that are costly to treat [27, 28]. Adequate clinical supervision may involve weekly meetings, ongoing education and training, case reviews, and support to PCs dealing with challenging clinical cases.

Our study examined a PrEP intervention across the SFDPH primary care clinics; therefore, our results may not be generalizable to other cities or specialty clinics. At time of writing this paper, the PrEP-OI study is ongoing; therefore, we do not currently have quantitative data on PrEP initiations or longer-term follow-up data on PrEP retention and persistence. We used CDC guidelines [18] and other publications [19] to develop the PrEP-Rx risk assessment; however, these questions were not validated across all patient populations. Finally, we do not have data from the perspective of the patients or other stakeholders regarding PrEP-OI implementation.

## Conclusion

In summary, our data reveal important findings regarding the benefits of a PrEP coordination program across the PrEP cascade of care, the need for ongoing education of providers, and measures to increase PrEP prescribing practices. Using this panel management task sharing strategy (including PrEP Coordinators and a web-based panel management tool), we believe we can increase PrEP uptake and persistence across clinical sites to further reduce HIV incidence and approach the goals for ending the HIV epidemic.

## Supporting information

S1 File (DOCX)

S2 File (DOCX)

## References

[1] Grant RM, Lama JR, Anderson PL, McMahan V, Liu AY, Vargas L, et al Preexposure Chemoprophylaxis for HIV Prevention in Men Who Have Sex with Men. New Engl J Med. 2010;363(27):2587–99. 10.1056/NEJMoa1011205 WOS:000285763700004. 21091279 PMC3079639

[2] Baeten JM, Donnell D, Ndase P, Mugo NR, Campbell JD, Wangisi J, et al Antiretroviral Prophylaxis for HIV Prevention in Heterosexual Men and Women. New Engl J Med. 2012;367(5):399–410. 10.1056/NEJMoa1108524 WOS:000307001900005. 22784037 PMC3770474

[3] Choopanya K, Martin M, Suntharasamai P, Sangkum U, Mock PA, Leethochawalit M, et al Antiretroviral prophylaxis for HIV infection in injecting drug users in Bangkok, Thailand (the Bangkok Tenofovir Study): a randomised, double-blind, placebo-controlled phase 3 trial. Lancet. 2013;381(9883):2083–90. 10.1016/S0140-6736(13)61127-7 . 23769234

[4] Harris NS, Johnson AS, Huang YA, Kern D, Fulton P, Smith DK, et al Vital Signs: Status of Human Immunodeficiency Virus Testing, Viral Suppression, and HIV Preexposure Prophylaxis—United States, 2013–2018. MMWR Morb Mortal Wkly Rep. 2019;68(48):1117–23. Epub 2019/12/06. 10.15585/mmwr.mm6848e1 31805031 PMC6897528

[5] Karris MY, Beekmann SE, Mehta SR, Anderson CM, Polgreen PM. Are We Prepped for Preexposure Prophylaxis (PrEP)? Provider Opinions on the Real-World Use of PrEP in the United States and Canada. Clin Infect Dis. 2014;58(5):704–12. 10.1093/cid/cit796 WOS:000331846700021. 24319083 PMC3922214

[6] Blumenthal J, Jain S, Krakower D, Sun X, Young J, Mayer K, et al Knowledge is Power! Increased Provider Knowledge Scores Regarding Pre-exposure Prophylaxis (PrEP) are Associated with Higher Rates of PrEP Prescription and Future Intent to Prescribe PrEP. Aids Behav. 2015;19(5):802–10. 10.1007/s10461-015-0996-z 25616837 PMC4417031

[7] Cohen SE, Liu AY, Bernstein KT, Philip S. Preparing for HIV Pre-Exposure Prophylaxis Lessons Learned from Post-Exposure Prophylaxis. American journal of preventive medicine. 2013;44(1):S80–S5. 10.1016/j.amepre.2012.09.036 WOS:000312528000005. 23253767 PMC3733170

[8] Krakower D, Ware N, Mitty JA, Maloney K, Mayer KH. HIV Providers' Perceived Barriers and Facilitators to Implementing Pre-exposure Prophylaxis in Care Settings: A Qualitative Study. Aids Behav. 2014;18(9):1712–21. 10.1007/s10461-014-0839-3 WOS:000340476000010. 24965676 PMC4127184

[9] Liu A, Cohen S, Follansbee S, Cohan D, Weber S, Sachdev D, et al Early experiences implementing pre-exposure prophylaxis (PrEP) for HIV prevention in San Francisco. Plos Med. 2014;11(3):e1001613 10.1371/journal.pmed.1001613 24595035 PMC3942317

[10] McBrien KA, Ivers N, Barnieh L, Bailey JJ, Lorenzetti DL, Nicholas D, et al Patient navigators for people with chronic disease: A systematic review. Plos One. 2018;13(2). ARTN e0191980 10.1371/journal.pone.0191980 WOS:000425554200011. 29462179 PMC5819768

[11] Steward WT, Koester KA, Guze MA, Kirby VB, Fuller SM, Moran ME, et al Practice transformations to optimize the delivery of HIV primary care in community healthcare settings in the United States: A program implementation study. Plos Med. 2020;17(3):e1003079 Epub 2020/03/28. 10.1371/journal.pmed.1003079 32214312 PMC7098549

[12] Schwartz MD, Jensen A, Wang B, Bennett K, Dembitzer A, Strauss S, et al Panel Management to Improve Smoking and Hypertension Outcomes by VA Primary Care Teams: A Cluster-Randomized Controlled Trial. Journal of general internal medicine. 2015;30(7):916–23. Epub 2015/02/11. 10.1007/s11606-015-3204-y 25666215 PMC4471025

[13] Chuang E, Ganti V, Alvi A, Yandrapu H, Dalal M. Implementing panel management for hypertension in a low-income, urban, primary care setting. Journal of primary care & community health. 2014;5(1):61–6. 10.1177/2150131913516497 . 24356533

[14] Pathela P, Jamison K, Blank S, Daskalakis D, Hedberg T, Borges C. The HIV Pre-exposure Prophylaxis (PrEP) Cascade at NYC Sexual Health Clinics: Navigation Is the Key to Uptake. J Acquir Immune Defic Syndr. 2020;83(4):357–64. Epub 2020/01/07. 10.1097/QAI.0000000000002274 . 31904700

[15] Spinelli MA, Scott HM, Vittinghoff E, Liu AY, Morehead-Gee A, Gonzalez R, et al A Panel Management and Patient Navigation Intervention Is Associated With Earlier PrEP Initiation in a Safety-Net Primary Care Health System. Jaids-J Acq Imm Def. 2018;79(3):347–51. 10.1097/Qai.0000000000001828 WOS:000457876800014. 30085955 PMC6185763

[16] Laborde ND, Kinley PM, Spinelli M, Vittinghoff E, Whitacre R, Scott HM, et al Understanding PrEP Persistence: Provider and Patient Perspectives. Aids Behav. 2020 Epub 2020/02/13. 10.1007/s10461-020-02807-3 . 32048078 PMC8054778

[17] Saberi P, Berrean B, Thomas S, Gandhi M, Scott H. A Simple Pre-Exposure Prophylaxis (PrEP) Optimization Intervention for Health Care Providers Prescribing PrEP: Pilot Study. JMIR Form Res. 2018;2(1). Epub 2019/01/15. 10.2196/formative.8623 30637375 PMC6325636

[18] Centers for Disease Control and Prevention: US Public Health Service. Preexposure prophylaxis for the prevention of HIV infection in the United States—2017 Update: a clinical practice guideline. 2018 [5/23/20]. Available from: https://www.cdc.gov/hiv/pdf/risk/prep/cdc-hiv-prep-guidelines-2017.pdf.

[19] Smith DK, Pan Y, Rose CE, Pals SL, Mehta SH, Kirk GD, et al A Brief Screening Tool to Assess the Risk of Contracting HIV Infection Among Active Injection Drug Users. J Addict Med. 2015;9(3):226–32. 10.1097/ADM.0000000000000123 WOS:000369710200010. 25961495 PMC4449303

[20] Stare A. Agile Project Management: A future approach to the management of projects?. Dynamic Relationships Management Journal. 2013.

[21] Ming K, Shrestha I, Vazquez A, Wendelborn J, Jimenez V, Lisha N, et al Improving the HIV PrEP continuum of care using an intervention for healthcare providers: a stepped-wedge study protocol. BMJ Open. 2020;10(7):e040734 Epub 2020/07/16. 10.1136/bmjopen-2020-040734 32665393 PMC7454188

[22] Coyne IT. Sampling in qualitative research. Purposeful and theoretical sampling; merging or clear boundaries? J Adv Nurs. 1997;26(3):623–30. Epub 1997/09/26. 10.1046/j.1365-2648.1997.t01-25-00999.x . 9378886

[23] Gale NK, Heath G, Cameron E, Rashid S, Redwood S. Using the framework method for the analysis of qualitative data in multi-disciplinary health research. Bmc Med Res Methodol. 2013;13 Artn 10.1186/1471-2288-13-117 WOS:000324780100001. PMC3848812 24047204

[24] Weiss G, Smith DK, Newman S, Wiener J, Kitlas A, Hoover KW. PrEP implementation by local health departments in US cities and counties: Findings from a 2015 assessment of local health departments. Plos One. 2018;13(7):e0200338 Epub 2018/07/26. 10.1371/journal.pone.0200338 30044820 PMC6059435

[25] Bernardo BM, Zhang XC, Hery CMB, Meadows RJ, Paskett ED. The efficacy and cost-effectiveness of patient navigation programs across the cancer continuum: A systematic review. Cancer-Am Cancer Soc. 2019;125(16):2747–61. 10.1002/cncr.32147 WOS:000477980700009. 31034604

[26] Fauci AS, Redfield RR, Sigounas G, Weahkee MD, Giroir BP. Ending the HIV Epidemic A Plan for the United States. Jama-J Am Med Assoc. 2019;321(9):844–5. 10.1001/jama.2019.1343 WOS:000460351600014. 30730529

[27] Kiran T, Kopp A, Moineddin R, Glazier RH. Longitudinal evaluation of physician payment reform and team-based care for chronic disease management and prevention. Can Med Assoc J. 2015;187(17):E494–E502. 10.1503/cmaj.150579 WOS:000364783000006. 26391722 PMC4646764

[28] Heintzman J, Cottrell E, Angier H, O'Malley J, Bailey S, Jacob L, et al Impact of Alternative Payment Methodology on Primary Care Visits and Scheduling. J Am Board Fam Med. 2019;32(4):539–49. 10.3122/jabfm.2019.04.180368 WOS:000475405600013. 31300574

