# Supplementary material for: “You can’t have a PrEP program without a PrEP Coordinator”: Implementation of a PrEP panel management intervention
Source: PLoS One. 2020 Oct 16;15(10):e0240745. doi: 10.1371/journal.pone.0240745 (PMC7567425; doi:10.1371/journal.pone.0240745)
Supplement: S1 File — (DOCX) [file pone.0240745.s001.docx]

**Provider and Staff Baseline Survey: PrEP-OI**

We are asking you to complete this survey so that we can collect demographic information about you and other Health Care Providers before PrEP-OI services (i.e., PrEP coordination and the PrEP-Rx tool) are launched. Additionally, we will be asking you questions about the clinic where you work, comfort with PrEP prescription, and clinical activities around HIV prevention. Your responses are confidential and will not be shared with the clinic leadership. All presentations or publications about the survey will present the overall results across all individuals who complete the survey. Your specific responses will not be reported. Please answer all questions to the best of your ability.

| **Name:** | | **Age:** | **Today’s Date:** |
| --- | --- | --- | --- |
| 1. | What is your gender identity?   1. Male 2. Female 3. Transgender Male 4. Transgender Female 5. Other, please specify: _________________ | | |
| 2. | How would you describe your racial identity?   1. American Indian / Alaska Native 2. Asian 3. Black or African American 4. Native Hawaiian or other Pacific Islander 5. Multiracial/Multicultural 6. White 7. Other, please specify: _________________ | | |
| 3. | Do you identify as Hispanic or Latino?   1. Yes 2. No | | |
| 4. | What is your Primary Profession/Role at this clinic? (Select one)   1. Nurse Practitioner 2. Pharmacist 3. Physician 4. Physician Assistant 5. Physician Intern/Resident 6. Physician Post-doctoral Fellow 7. Registered Nurse 8. Other, please specify: _________________________ | | |
| 5. | What is your primary specialty?   1. Family Medicine 2. Infectious Diseases 3. Internal Medicine 4. Other, please specify: ____________________________ 5. Not Applicable | | |

| 6. | At which clinic(s) do you currently work? Please check all that apply   - 3rd Street Youth Center and Clinic - Balboa Teen Health Center - Castro Mission Health Center - Chinatown Public Health Center - Cole Street Youth Clinic - Dimensions Clinic - Family Health Center - Larkin Street Youth Clinic - Maxine Hall Health Center - New Generation Health Center - Potrero Hill Health Center - Richard H. Fine People’s Clinic (1M) - Silver Avenue Family Health Center - Southeast Health Center - Tom Waddell Urban Health Center - Other, please specify: ____________________________ |
| --- | --- |
| 7. | How many years have you been providing direct patient care?  Please specify the number of years: ______________________ years |
| 8. | Do you provide services in any languages other than English?   1. Yes 2. No   If “yes”, Please specify the language(s): ______________________ |
| 9. | Thinking across all clinics you work at, approximately how many patients are currently in your panel(s)/under your care?  Please specify the number of patients: _____________________ patients |
| 10. | Do you provide services directly to clients/patients living with HIV?   1. Yes 2. No |
| 11. | If answered ‘yes’ to last question, how many patients in your panel are currently receiving HIV treatment (i.e., antiretroviral therapy)?  Please specify the number of patients: ______________________ patients |
| 12. | Have you ever prescribed PrEP (i.e., Truvada) for a patient?   1. Yes 2. No |
| 13. | Have you ever referred a patient for PrEP (e.g., to a PrEP provider, PrEP Coordinator, or HIV clinic)?   1. Yes 2. No |
| 14. | How many patients in your panel are currently receiving PrEP (Truvada) as HIV prevention?  Please Specify the number of patients: _____________________ patients |
| 15. | Are you willing to prescribe PrEP for adolescents (13-17 years):   1. Yes 2. No 3. Not sure 4. Not applicable |
| 16. | Are you willing to prescribe PrEP to adults (≥18 years):   1. Yes 2. No 3. Not sure |

17. In your practice, who currently provides the following types of PrEP services?

|  | **Me** | **Nurse** | **Counselor** | **Social worker** | **Off-site clinic staff** | **PrEP Coordinator** | **No one** |
| --- | --- | --- | --- | --- | --- | --- | --- |
| Sexual risk reduction counseling |  |  |  |  |  |  |  |
| PrEP adherence counseling |  |  |  |  |  |  |  |
| Laboratory tests and monitoring |  |  |  |  |  |  |  |

18. In the past year, how often have you done the following with patients?

|  | **Never** | **Rarely** | **Occasionally** | **Often** | **Always** |
| --- | --- | --- | --- | --- | --- |
| Initiated a conversation about PrEP |  |  |  |  |  |
| Asked about sexual partner(s) |  |  |  |  |  |
| Asked about sexual partners' HIV status |  |  |  |  |  |
| Asked about sex practices |  |  |  |  |  |
| Asked about condom use |  |  |  |  |  |
| Offered HIV testing to patients who do not engage in high-risk behaviors |  |  |  |  |  |
| Offered HIV testing to patients who engage in high-risk behaviors |  |  |  |  |  |

19. Please indicate your degree of agreement with the following statements:

|  | **Strongly disagree** | **Disagree** | **Neutral** | **Agree** | **Strongly agree** |
| --- | --- | --- | --- | --- | --- |
| I am confident that I or someone in my clinic can identify individuals at-risk for HIV infection |  |  |  |  |  |
| I am confident that I or someone in my clinic can provide an HIV test every 3 months for at-risk individuals on PrEP |  |  |  |  |  |
| I am confident that I or someone in my clinic can follow up at-risk patients every 3 months to monitor side effects from PrEP |  |  |  |  |  |
| I am confident that I or someone in my clinic can provide risk reduction and medication-adherence counseling to patients on PrEP |  |  |  |  |  |

20. Assuming no medical contraindications to emtricitabine/tenofovir, to which of the following HIV-uninfected patients would you seriously consider prescribing PrEP? (Circle all that apply)

|  | **Yes** | **No** |
| --- | --- | --- |
| Men who have sex with men (MSM) in a non-monogamous relationship with an HIV(+) male partner |  |  |
| Women in a non-monogamous relationship with an HIV(+) male partner |  |  |
| Men or women in a serodiscordant relationship with an HIV(+) male partner, but only if they were monogamous |  |  |
| MSM with multiple sex partners |  |  |
| MSM with inconsistent condom use for anal sex as a top (insertive partner) |  |  |
| MSM with inconsistent condom use for anal sex as a bottom (receptive partner) |  |  |
| Women with multiple sex partners |  |  |
| Transgender women (i.e. male-to-female) having sex with men |  |  |

21. What concerns do you have about PrEP as an HIV prevention tool (Circle all that apply)

|  | **Yes** | **No** |
| --- | --- | --- |
| Concerns about patient adherence to the PrEP regimen |  |  |
| Concerns about development of HIV resistance |  |  |
| Concerns about patients increasing risky sexual behaviors when taking PrEP |  |  |
| Concerns about cost |  |  |
| Concerns about toxicity (e.g., kidney, bone) |  |  |
| Concerns about side effects (e.g., nausea) |  |  |
| Concerns about provider reimbursement or workload |  |  |
| Other: ______________________________________________________ |  |  |
