# Supplementary material for: “You can’t have a PrEP program without a PrEP Coordinator”: Implementation of a PrEP panel management intervention
Source: PLoS One. 2020 Oct 16;15(10):e0240745. doi: 10.1371/journal.pone.0240745 (PMC7567425; doi:10.1371/journal.pone.0240745)
Supplement: S2 File — (DOCX) [file pone.0240745.s002.docx]

**Improving the HIV PrEP Cascade using an Intervention for Healthcare Providers**

**Qualitative In-Depth Interview Guide for Healthcare Providers**

Thank you so much for agreeing to participate in our study. As you know from our conversation when we were completing the consent form, the purpose of our overall study is to examine the use of a our intervention (i.e., PrEP Coordination plus our online tool, PrEP-Rx) to educate health care providers on HIV PrEP, assist providers in appropriate targeting of at risk patients, and initiating and continuing PrEP in appropriate patients.

In our conversation today, I’d like to hear about your experiences during the course of our study and your thoughts about potential approaches to improving this experience. With your help, we will refine our intervention and make it more generalizable.

Participation in this interview is totally voluntary. If there are any questions that you don’t want to answer, that is fine. It is also fine if you decide at any time that you want to stop the interview.

I’ll be making an audio-recording of the interview, and we’ll transcribe it. Your name will not be linked to the interview, and the recordings will be destroyed at the end of the study.

Are you ready to begin? (*Start audio-recorder*)

**RECORD!!!**

**Questions:**

1. Prior to our study, can you please tell me a bit about your experiences with PrEP initiation and/or monitoring?
   1. How many patients did you see on PrEP? How did you follow-up with them?
   2. What hurdles did you face? What hurdles do you think the patients may have faced?
2. Can you please tell me about your experiences initiating and/or monitoring PrEP since our study began?
   1. What has changed?
   2. What benefits have you noted?
   3. What challenges have you noted?
3. How many patients do you currently have on PrEP? How do you follow-up with them?
   1. At which steps of the PrEP cascade of care (starting from education to patient all the way to quarterly monitoring) do you typically involved the PrEP Coordinator?
   2. What changes have you notice with regard to time to PrEP initiation? How many days until PrEP start before PrEP Coordinator? How many days now?
4. How easy or difficult is it to communicate with the PrEP Coordinator?
   1. On average, how many times a week do you communicate with them?
   2. How quickly does the PrEP Coordinator usually get back to you?
5. How do you think the presence and work of the PrEP Coordinator has changed perceptions on PrEP or PrEP prescribing and management practices in your clinic?
6. How do you think the presence and work of the PrEP Coordinator has changed practices around STI testing (e.g., self-swabbing) or provision of PEP in your clinic?
7. Overall, how do you think we can modify this PrEP service to make it as useful as possible?
8. In general, given that PrEP prescribing is still low across SFDPH clinics, what other measures do you think would be helpful to increase PrEP prescribing in your clinic?
9. When the FDA approves new PrEP formulations (e.g., injections, implants, vaginal rings, etc.), how do you think providers can best be supported to prescribe these formulations?
10. Is there anything that we didn’t touch upon that you’d like to discuss with regard to the study?

**Thank you so much for meeting with us today! We really appreciate your time!**
